# Attenuation Effect of Radiofrequency Irradiation on UV-B-Induced Skin Pigmentation by Decreasing Melanin Synthesis and through Upregulation of Heat Shock Protein 70

**DOI:** 10.3390/molecules26247648

**Published:** 2021-12-17

**Authors:** Hyoung Moon Kim, Seyeon Oh, Chang Hu Choi, Jin Young Yang, Sunggeun Kim, Donghwan Kang, Kuk Hui Son, Kyunghee Byun

**Affiliations:** 1 Department of Anatomy & Cell Biology, Gachon University College of Medicine, Incheon 21936, Korea; drmac12@me.com; 2 Functional Cellular Networks Laboratory, Department of Medicine, Graduate School and Lee Gil Ya Cancer and Diabetes Institute, College of Medicine, Gachon University, Incheon 21999, Korea; seyeon8965@gmail.com (S.O.); roswellgirl111@gmail.com (J.Y.Y.); 3 Department of Thoracic and Cardiovascular Surgery, Gachon University Gil Medical Center, Gachon University, Incheon 21565, Korea; cch624@gilhospital.com; 4 Jeisys Medical Inc., Seoul 08501, Korea; sunggeun.kim@jeisys.com (S.K.); kang@jeisys.com (D.K.)

**Keywords:** melanogenesis, radiofrequency microneedling, HSP70, skin pigmentation

## Abstract

Excess melanin deposition in the skin causes cosmetic problems. HSP70 upregulation decreases microphthalmia-associated transcription factor (MITF) expression, which eventually decreases tyrosinase activity and melanogenesis. Ultraviolet (UV) radiation upregulates p53, which increases the melanocortin receptor (MC1R) and MITF. Furthermore, HSP70 decreases p53 and radiofrequency irradiation (RF) increases HSP70. We evaluated whether RF increased HSP70 and decreased p53, consequently decreasing the MITF/tyrosinase pathway and melanogenesis in UV-B radiated animal skin. Various RF combinations with 50, 100, and 150 ms and 5, 10, and 15 W were performed on the UV-B radiated mouse skin every 2 d for 28 d. When RF was performed with 100 ms/10 W, melanin deposition, evaluated by Fontana–Masson staining, decreased without skin crust formation in the UV-B radiated skin. Thus, we evaluated the effect of RF on decreasing melanogenesis in the HEMn and UV-B radiated skin at a setting of 100 ms/10 W. HSP70 expression was decreased in the UV-B radiated skin but was increased by RF. The expression of p53, MC1R, and MITF increased in the UV-B radiated skin but was decreased by RF. The expression of p53, MC1R, and MITF increased in the α-MSH treated HEMn but was decreased by RF. The decreasing effects of RF on p53, MC1R, CREB and MITF were higher than those of HSP70-overexpressed HEMn. The decreasing effect of RF on p53, MC1R, CREB, and MITF disappeared in the HSP70-silenced HEMn. MC1R, CREB, and MITF were not significantly decreased by the p53 inhibitor in α-MSH treated HEMn. RF induced a greater decrease in MC1R, CREB, and MITF than the p53 inhibitor. Therefore, RF may have decreased melanin synthesis by increasing HSP70 and decreasing p53, thus decreasing MC1R/CREB/MITF and tyrosinase activity.

## 1. Introduction

The skin is a barrier organ which handles environmental stress and has the ability to maintain cutaneous homeostasis [1]. By the absorption of ultraviolet (UV) light in the skin, the defense mechanisms for decreasing the disruption of skin integrity and homeostasis are initiated [1]. Furthermore, UV leads to increased activity of the local neuroendocrine system which upregulates production of various cytokines, corticotropin-releasing hormone, urocortins, proopiomelanocortin peptides, and enkephalins. Those effectors show systemic effects by releasing themselves into circulation and lead to the activation of the central hypothalamic-pituitary-adrenal axis, immunosuppression, and opioidogenic effects [1].

Melanogenesis is one of protective mechanisms which is triggered by UV. Melanogenesis is a complex regulatory process which is controlled by various pathways, such as receptor-dependent or receptor-independent mechanisms, in hormonal, autocrine, paracrine, or intracrine manners [2,3]. The most well-known positive inducer of melanogenesis is the melanocortin receptor (MC1R) and its ligands such as adrenocorticotropic hormone (ACTH) and α-melanocyte-stimulating hormone (α-MSH) [2].

However, skin hyperpigmentation resulting from increased melanin synthesis and deposition induces various cosmetic problems, including post-inflammatory hyperpigmentation, freckles, senile lentigines, dots, and hyperpigmentations [4]. Hyperpigmentation progresses in three stages in the epidermis: melanocyte proliferation, melanin production (melanogenesis) through the activation of tyrosinase, and melanosome transfer from melanocytes to keratinocytes [5,6,7,8]. However, hyperpigmentation is mainly induced by melanin accumulation in keratinocytes [9].

UV light irradiation enhances melanogenesis by increasing α-MSH [10]. UV light increases p53 activation, which leads to increased proopiomelanocortin (POMC) in keratinocytes, eventually increasing α-MSH production [10]. Furthermore, the microphthalmia-associated transcription factor (MITF) is activated by binding α-MSH to an MC1R, which is a G-protein-coupled receptor [10,11,12,13,14]. Thus, MITF is the main regulator of melanogenesis, modulating the survival, proliferation, and growth of melanocytes [11].

Melanogenesis is a consequent process of melanosomal maturation, consisting of four stages (stage I–IV). In the melanocyte, the structure of a stage I melanosome looks like a maturing multivesicular body, and it has fibrils consisting of melanosomal matrix protein 17 (PMEL17) and intralumenal vesicles [15,16,17]. A stage II melanosome changes into an ellipsoidal form by reorganization of the fibrils [18,19,20]. There are no melanin deposits in stage I and II melanosomes. Thus, stage I and II melanosomes are called premelanosomes. In stage III melanosomes, melanin is produced by tyrosinase, tyrosinase-related protein 1 (TYRP1), and TYRP2, then deposited on the PMEL fibrils [18,19,20]. Finally, stage IV melanosomes are filled with melanin and appear completely dark [18,19,20]. Increased MITF activity leads to increased tyrosinase expression and TYRP1, which eventually increases melanin biosynthesis [21,22]. Moreover, numerous membrane-trafficking proteins, such as the small Ras-like GTPases/Rab protein family members, RAB-27A, RAB-32, and RAB-38, are involved in melanosome maturation [23]. RAB-27A expression, which is essential for transporting mature melanosome to keratinocytes, is also increased by MITF [24,25].

By exposing cells to stresses, such as heat or UV irradiation, stress proteins for decreasing injuries from stress, such as heat shock protein (HSP), are activated. For example, HSP70, as a stress protein, leads to the refolding or degradation of denatured proteins which result from stress including reactive oxygen species (ROS) [26]. It has been reported that transgenic mice expressing HSP70 exhibited decreased UV-induced skin damage that resulted in decreased epidermal apoptosis, DNA damage, and wrinkles in the skin [27,28]. Moreover, HSP70 overexpression leads to decreased melanogenesis in mouse melanoma (B16) cells [29]. Melanogenesis by UV radiation is also decreased in transgenic mice expressing HSP70 [29]. The effects of HSP70 on decreasing skin damage or melanogenesis are mediated by the cytoprotective or anti-inflammatory role of HSP70 [27,28]. Moreover, HSP70 directly binds to MITF, which sequentially leads to decreased tyrosinase expression by inhibiting the specific binding sites of MITF to the promoter of the tyrosinase gene [29]. Meanwhile, p53 is upregulated by various stresses, such as UV irradiation, aging, and inflammation [30]. Various HSPs, such as HSP70, HSP90, and HSP40, are involved in p53 regulation [31]. For example, HSP70 maintains the stability of wild-type p53 at higher temperatures to support its DNA-binding [32,33,34]. At the same time, HSP70 is also involved in sequestering p53 in the cytoplasm, which negatively affects its stability [35,36]. In cancer, HSP70 provides stability to mutant p53 [37]. Paradoxically, HSP70 also leads to the degradation of mutant p53 [38,39].

Our group has previously shown that radiofrequency (RF) irradiation decreases UV-B induced skin pigmentation by decreasing pro-inflammatory cytokines, such as interleukin (IL)-6 or IL-8, which helps to decrease skin inflammation [40]. Moreover, RF decreases skin pigmentation by increasing melanosomal autophagy in UV-B radiated animal skin [41]. RF generates heat through a high-frequency electric current. The electrical current increases the temperature of the deep dermis, fibrous septae, and fat [42]. The heat produced by RF in the dermis leads to the denaturation and shorting of collagen fibers, which eventually increases the activity of fibroblasts and collagen synthesis [42,43,44].

It has also been reported that RF increases collagen synthesis by generating heat, leading to increased expression of HSP27, HSP47, and HSP70 [45]. Although RF increased HSPs by generating heat, it has not been fully revealed whether RF could decrease skin pigmentation by increasing HSP70. Therefore, we hypothesized that RF increases HSP70, consequently decreasing the MITF/tyrosinase pathway in UV-B radiated animal skin. Furthermore, increased HSP70 might be involved in p53 modulation, which decreases MITF and melanogenesis.

## 2. Results

### 2.1. RF Decreased Melanin Accumulation in the UV-B Radiated Skin

**Figure 1:**
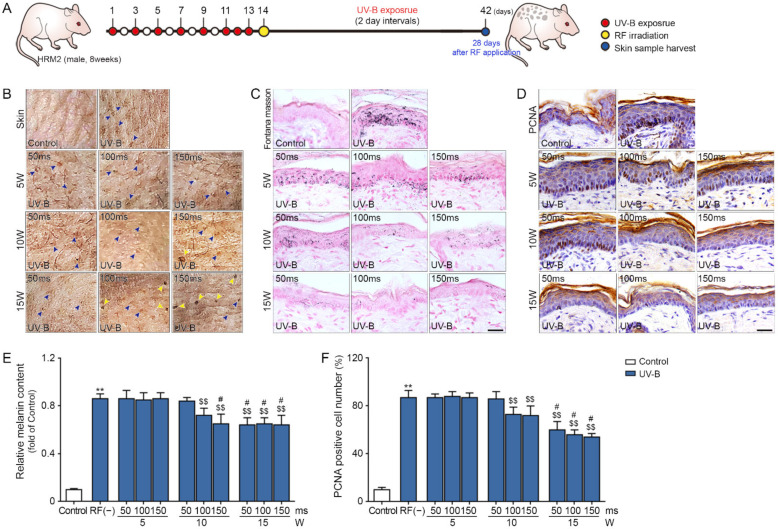
Decreased melanin accumulation due to RF in the UV-B exposed skin. (**A**) Diagram of the animal model used to validate the melanin reduction mechanism. (**B**) Change in animal skin according to power of RF irradiation. (**C**,**E**) Change in Fontana–Masson staining of skin tissue according to power of RF irradiation. (**D**,**F**) Change in PCNA positive skin tissue cells according to power of RF irradiation. PCNA, proliferating cell nuclear antigen; RF, radiofrequency; UV-B, ultraviolet-B; **: *p* < 0.01 vs. control; $$: *p* < 0.01 vs. UV-B/RF (−); #: *p* < 0.05 vs. UV-B/RF 10 W/100 ms.

RF irradiation at 50, 100, or 150 ms and 5, 10, or 15 W was performed on UV-B radiated mouse skin every 2 d for 28 d (Figure 1A). The skin pigmentation was increased by UV-B radiation, and RF decreased skin pigmentation (Figure 1B). In addition, the epidermal crust was shown when RF was radiated by 150 ms/10 W, 50 ms/15 W, 100 ms/15 W, and 150 ms/15 W.

Fontana–Masson staining showed that melanin deposition in the UV-B group was significantly greater than in the control groups (Figure 1C,E). RF radiation at 50, 100, and 150 ms with 5 W showed no significant decrease in melanin accumulation compared with the UV-B group. Additionally, there was no difference in melanin accumulation between 50 ms of RF with the 10 W radiated group and the UV-B groups. An RF of 100 (1.21 fold) and 150 ms (1.32 fold) with 10 W significantly decreased melanin accumulation compared to the UV-B group. Furthermore, RFs of 50, 100, and 150 ms with 15 W (1.34, 1.32, and 1.34-fold, respectively) significantly decreased melanin accumulation compared to the UV-B group. Since the skin crust was shown from 150 ms/10 W, we thought the proper RF irradiation could be 100 ms/10 W, which decreased melanin deposition without skin injury. Statistical differences between the RF radiation groups of various irradiation settings and RF of 100 ms/10 W were compared. Melanin accumulation from RFs of 150 ms/10 W, 50 ms/15 W, 100 ms/15 W, and 150 ms/15 W were significantly lower than that of the 100 ms/10 W group.

The PCNA (proliferating cell nuclear antigen) stain showed that PCNA-positively-stained cells in the epidermis were significantly increased in the UV-B irradiation group compared to the control group (Figure 1D,F). PCNA-positive cells at 50, 100, and 150 ms with 5 W and the 50 ms/10 W group were not significantly different from the UV-B irradiation groups. However, PCNA-positive cells at 100 and 150 ms with 10 W and 50, 100, and 150 ms with 15 W were significantly lower than the UV-B irradiation group.

### 2.2. RF Decreased Melanin Synthesis and Formation of Stage IV Melanosome

**Figure 2:**
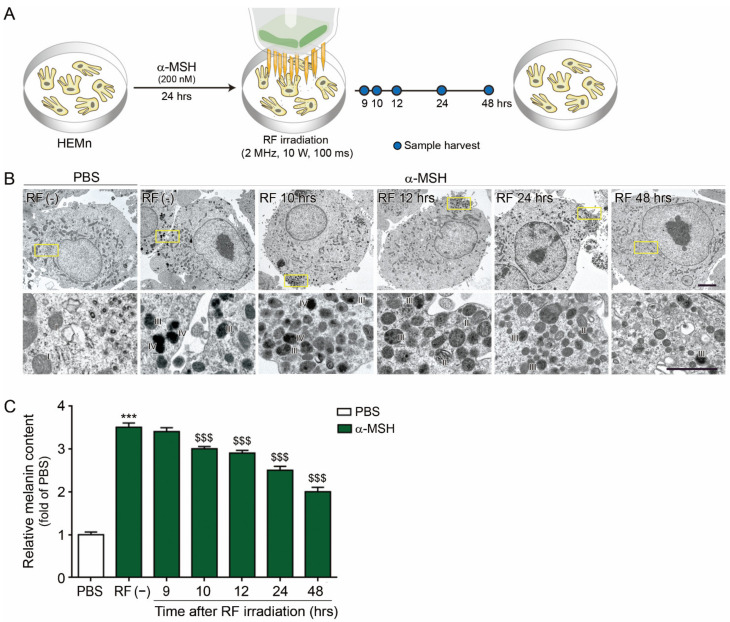
Attenuated effects of melanin synthesis and formation of stage IV melanosomes by RF. (**A**) Diagram of the in vitro model used to validate the melanin reduction mechanism. (**B**) Change in melanosome maturation with time after RF irradiation in TEM. (**C**) Change in the melanin content of α-MSH-treated HEMn. α-MSH, α-melanocyte stimulating hormone; HEMn, human epidermal melanocyte cells; RF, radiofrequency; TEM, transmission electron microscope; UV-B, ultraviolet-B; ***: *p* < 0.001 vs. PBS; $$$: *p* < 0.001 vs. α-MSH/RF (−).

We evaluated the effect of RF on decreasing melanogenesis with human epidermal melanocyte cells (HEMn), which stimulated melanogenesis by α-MSH treatment. RF was irradiated on α-MSH treated HEMn by the 2 MHz, 100 ms, and 10 W condition (Figure 2A).

TEM showed that HEMn mainly contained stage I melanosomes without α-MSH treatment (Figure 2B). However, α-MSH treated HEMn showed stage III or IV melanosomes. Stage IV melanosomes began disappearing 12 h after RF irradiation. However, at 48 h after RF, stage II or III melanosomes were mainly seen in the α-MSH treated HEMn (Figure 2B). The melanin content was significantly decreased 10 h after RF irradiation (Figure 2C).

### 2.3. RF Increased the Expression of HSP70 and Decreased the Expression of p53, MC1R, and MITF

**Figure 3:**
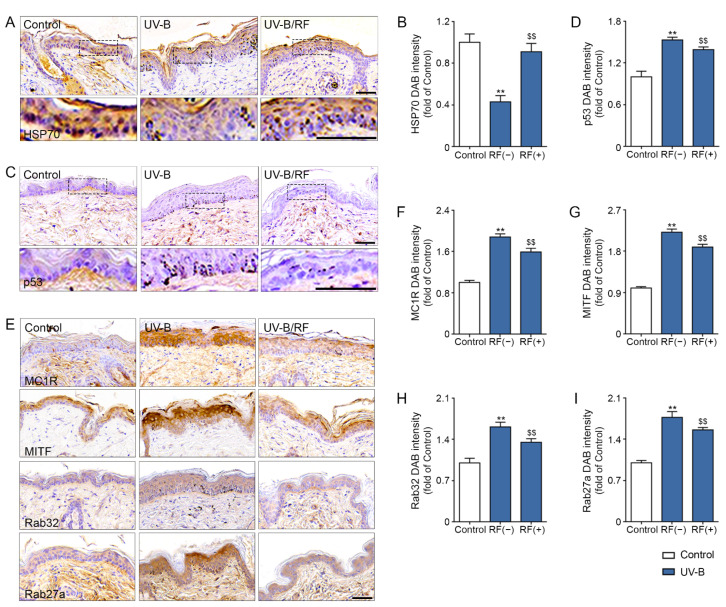
Upregulation of HSP70 and downregulation of p53, MC1R, and MITF by RF in UV-B exposed skin (**A**–**D**). HSP70 and p53 expression levels of UV-B exposed mouse skin were evaluated immunohistochemically (scale bar = 100 μm). (**E**–**I**) MC1R (fist lane of **E**,**F**), MITF (second lane of **E**,**G**), Rab32 (third lane of **E**,**H**), and Rab27a (fourth lane of **E**,**I**) expression levels of UV-B exposed mouse skins were evaluated immunohistochemically (scale bar = 50 μm). HSP70, 70 kilodalton heat shock proteins; MC1R, melanocortin 1 receptor; MITF, microphthalmia-associated transcription factor; Rab32, ras-related protein Rab-32; Rab27a, ras-related protein Rab-27a; RF, radiofrequency; UV-B, ultraviolet-B; **: *p* < 0.01 vs. control; $$: *p* < 0.01 vs. UV-B/RF (−).

HSP70 expression in the UV-B radiated mouse skins was significantly decreased compared to that of the control mice. However, it was significantly increased by RF irradiation (2 MHz, 100 ms, and 10 W; Figure 3A,B).

The expression of p53 in the UV-B radiated mouse skins was significantly increased compared to that of the control mice. Conversely, it was significantly decreased by RF irradiation (Figure 3C,D).

The expressions of MC1R, MITF, RAB32, and RAB27a in UV-B radiated animal skin significantly increased compared to those of the control mice. However, these were significantly decreased by RF irradiation (Figure 3E–I).

### 2.4. HSP70 Is Involved in Decreasing Melanin Synthesis

To evaluate whether the signal pathway of HSP70 or p53 modulates melanogenesis, RF was irradiated to HSP70-overexpressed HEMn, HSP70-silenced HEMn or p53 inhibitor-treated HEMn cells.

**Figure 4:**
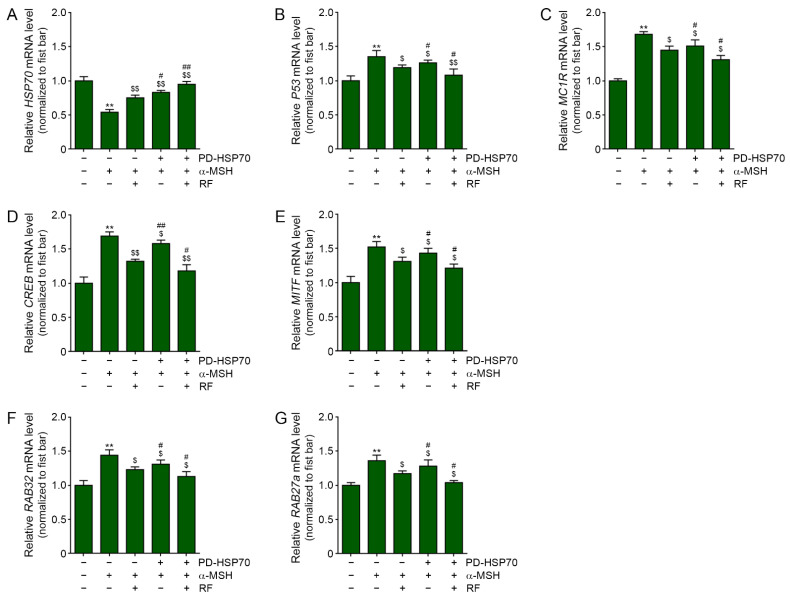
Effects of RF effect on decreasing melanogenesis in HSP70-overexpressed HEMn cells (**A**–**G**). HSP70 (**A**), p53 (**B**), MC1R (**C**), CREB (**D**), MITF (**E**), RAB32 (**F**) and RAB27a (**G**) mRNA levels of α-MSH and PD-HSP70 or untreated melanocytes were evaluated by qRT-PCR. HSP70, 70 kilodalton heat shock proteins; MC1R, melanocortin 1 receptor; MITF, microphthalmia-associated transcription factor; Rab32, ras-related protein Rab-32; Rab27a, ras-related protein Rab-27a; RF, radiofrequency; UV-B, ultraviolet-B; **: *p* < 0.01 vs. PBS; $: *p* < 0.05, $$: *p* < 0.01 vs. α-MSH/RF (−); #: *p* < 0.05, ##: *p* < 0.01 vs. α-MSH/RF (+).

First, we evaluated the effects of RF on decreasing the expression of p53, MC1R, element-binding protein (CREB), MITF, RAB32, and RAB27a in HSP70-overexpressed HEMn cells (Figure 4).

The expression of HSP70 was significantly decreased when normal HEMn cells were treated with α-MSH. It was increased by RF or HSP70 overexpression. HSP70 expression was lower in the RF radiated HEMn cells than that in HSP70-overexpressed HEMn cells. When RF was radiated to HSP70-overexpressed HEMn cells, the expression of HSP70 was highest (Figure 4A). The expression of p53 was increased by α-MSH treatment in normal HEMn cells. It was decreased by RF or HSP70 overexpression. The decreasing effect of RF on p53 was higher in the RF radiated HEMn cells than in the HSP70-overexpressed HEMn cells. The expression of p53 was lowest when RF was radiated to HSP70-overexpressed HEMn cells (Figure 4B). MC1R expression was increased by α-MSH treatment to normal HEMn cells. It was decreased by RF or HSP70 overexpression. The decreasing effect of RF on MC1R was higher in the RF radiated HEMn cells than in the HSP70-overexpressed HEMn cells. It was lowest when RF was radiated to HSP70-overexpressed HEMn cells (Figure 4C). CREB expression was increased by α-MSH treatment of normal HEMn cells. It was decreased by RF or HSP70 overexpression. The decreasing effect of RF on CREB was higher in the RF radiated HEMn cells than in the HSP70-overexpressed HEMn cells. It was lowest when RF was radiated to HSP70-overexpressed HEMn cells (Figure 4D). MITF expression was increased by α-MSH treatment of normal HEMn cells. It was decreased by RF or HSP70 overexpression. The decreasing effect of RF on MITF was higher in the RF radiated HEMn cells than in the HSP70-overexpressed HEMn cells. It was lowest when RF was radiated to HSP70-overexpressed HEMn cells (Figure 4E). RAB32 and RAB27a expressions were increased by α-MSH treated to normal HEMn cells. These were decreased by RF or HSP70 overexpression. The decreasing effects of RF on RAB32 and RAB27a were higher in the RF radiated HEMn cells than in the HSP70-overexpressed HEMn cells (Figure 4F,G). These were lowest when RF was radiated to HSP70-overexpressed HEMn cells.

RF showed a decreasing effect on the expression of p53, MC1R, CREB, MITF, RAB32, and RAB27a that was greater than the effects of HSP70 overexpression.

**Figure 5:**
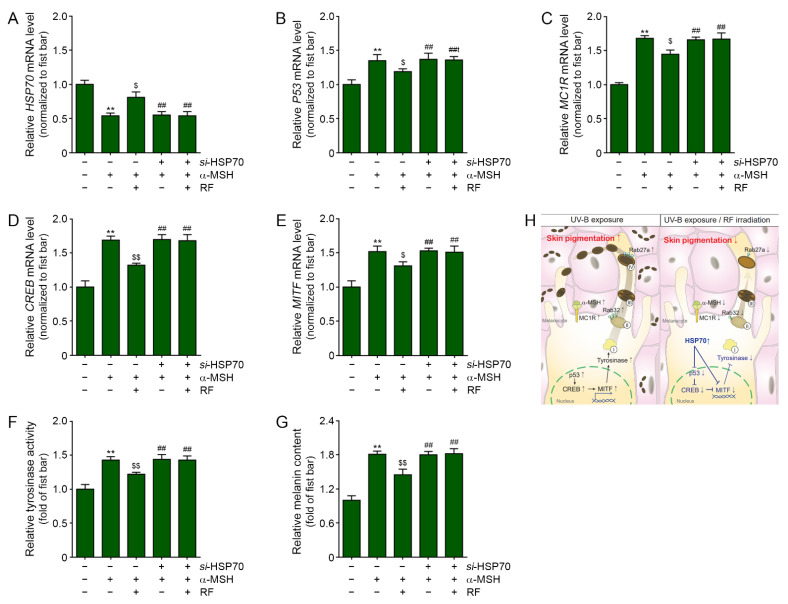
Effects of RF on decreasing melanogenesis in HSP70-silenced HEMn cells (**A**–**E**). HSP70 (**A**), p53 (**B**), MC1R (**C**), CREB (**D**), and MITF (**E**) mRNA levels of α-MSH and si-HSP70 or untreated melanocytes were evaluated using qRT-PCR. (**F**) Relative tyrosinase activity of α-MSH and si-HSP70 treated melanocytes were evaluated by ELISA. (**G**) Relative melanin content of α-MSH and si-HSP70 treated melanocytes were evaluated by melanin content assay. (**H**) Summary of this study. HSP70, 70 kilodalton heat shock proteins; MC1R, melanocortin 1 receptor; MITF, microphthalmia-associated transcription factor; Rab32, ras-related protein Rab-32; Rab27a, ras-related protein Rab-27a; RF, radiofrequency; UV-B, ultraviolet-B; **: *p* < 0.01 vs. PBS; ##: *p* < 0.01 vs. α-MSH; $: *p* < 0.05 vs. α-MSH/RF; $$: *p* < 0.01 vs. α-MSH/RF.

Next, we evaluated the expression of the signal pathways involved in melanogenesis in HSP70-silenced HEMn cells (Figure 5). Treating normal HEMn with α-MSH decreased HSP70 expression. The decreased HSP70 expression was then increased by RF radiation. However, the increasing effect of RF on HSP70 disappeared in the HSP-silenced group (Figure 5A). The expression of p53 was increased by α-MSH treatment and it was decreased by RF radiation in normal HEMn cells. However, the decreasing effect of RF on p53 expression disappeared in the HSP70-silenced group (Figure 5B). The expressions of MC1R/CREB/MITF were increased by α-MSH treatment and decreased by RF in the HEMn cells. However, the decreasing effects of RF disappeared in the HSP70-silenced group (Figure 5C–E).

Tyrosinase activity was increased by α-MSH treatment and decreased by RF in the normal HEMn cells. However, the decreasing effect of RF on tyrosinase activity disappeared in the HSP70-sileneced group (Figure 5F). The melanin content increased by α-MSH treatment and was decreased by RF in the normal HEMn cells. In HSP70-silenced HEMn cells, the decreasing effect of RF on melanin content disappeared (Figure 5G). 

These results show that HSP70, which is upregulated by RF, is involved in decreasing p53/MC1R/CREB/MITF and tyrosinase activity.

Next, we evaluated the expression of HSP70, MC1R, CREB, MITF in p53 inhibitor-treated HEMn cells (Figure S1).

HSP70 expression, which was decreased by α-MSH treatment, was increased by RF. There was no increasing effect of HSP70 expression when HEMn cells were treated with a p53 inhibitor. However, RF restored HSP70 expression in the α-MSH and p53 inhibitor treated HEMn cells (Figure S1A). The expression of p53 was increased by α-MSH treatment and decreased by RF. The decreasing effect of RF on p53 expression was lower than p53 inhibitor treatment or p53 inhibitor treatment with RF (Figure S1B). MC1R expression was increased by α-MSH treatment and decreased by RF. MC1R expression in the RF radiated HEMn cells was lower than that of p53 inhibitor treated HEMn cells, however it was similar with RF/p53 inhibitor treated HEMn cells (Figure S1C). CREB expression was increased by α-MSH and decreased by RF. CREB expression of RF radiated HEMn cells was lower than that of p53 inhibitor treated HEMn cells; however, it was similar to that of RF/p53 treated HEMn cells. CREB expression was not significantly different between α-MSH treated HEMn cells and α-MSH/p53 inhibitor treated HEMn cells (Figure S1D). MITF expression was increased by α-MSH and was decreased by RF. The MITF expression of RF radiated HEMn cells was lower than that of p53 inhibitor treated HEMn, however it was higher than that of RF/p53 inhibitor treated HEMn cells. MITF expression was not significantly different between α-MSH treated HEMn cells and α-MSH/p53 inhibitor-treated HEMn cells (Figure S1E).

Tyrosinase activity was increased by α-MSH and was decreased by RF. The tyrosinase activity of RF radiated HEMn cells was lower than that of p53 inhibitor treated HEMn cells. However, it was similar to that of RF/p53 inhibitor treated HEMn cells (Figure S1F). Melanin content was increased by α-MSH and it was decreased by RF. The tyrosinase activity of RF-radiated HEMn cells was lower than that of p53 inhibitor treated HEMn cells. However, it was similar to that of RF/p53 inhibitor treated HEMn cells (Figure S1G).

## 3. Discussion

UV irradiation induces melanogenesis in the basal layer of the epidermis. Newly synthesized melanin granules by UV radiation are moved to the basal or suprabasal epidermal keratinocyte and form a supranuclear cap, which plays a role in protecting the skin from UV radiation [46,47]. However, hyperpigmentation could lead to unfavorable cosmetic problems.

In the initial steps of melanogenesis, tyrosinase plays an essential role in catalyzing tyrosine to pheomelanin or eumelanin. Pheomelanin and eumelanin are synthesized by tyrosinase, which catalyzes the hydroxylation of L-tyrosine to 3,4-dihydroxy-L-phenylalanine (L-DOPA) [48,49,50]. Moreover, L-DOPA is oxidized into dopaquinone by tyrosinase. When cysteine is presented, dopaquinone is changed to yellow or red pheomelanin by oxidation and polymerization. In the absence of cysteine, dopaquinone changes into DOPAchrome, which changes into brown or black eumelanin due to the action of TRP 1 and TRP2 [51,52,53].

MC1R, expressed on melanocytes, controls melanogenesis by binding its ligand (α-MSH) [54,55,56,57]. Activated MC1R increases cAMP synthesis through G-protein and sequentially upregulates the phosphorylation of the CREB transcription factor, which increases its responsiveness to cAMP. In addition, upregulated CREB leads to the activation of MITF, which is essential for melanocyte and melanogenesis differentiation [58,59]. Furthermore, α-MSH secretion in keratinocytes by UV radiation was positively associated with melanogenesis [60]. Additionally, UV radiation increased other melanogenic proteins, such as p53 and POMC [61].

HSP70 expression in the skin is increased by various stresses, including heat [62,63]. As a protection mechanism, HSP expression in keratinocytes is also increased by UV irradiation [63,64,65,66,67]. Moreover, HSP70 overexpression in keratinocytes and melanocytes protected cells against injury from UV in in vitro and in vivo models [62,67,68,69,70,71]. HSP70 null mice showed susceptibility to UV-induced skin damage [71].

Additionally, the heat treatment of melanoma cells leads to decreasing melanin synthesis. The mechanism that decreases melanin synthesis by heat has been suggested to decrease ERK activation and inhibit protein phosphatase 2A [72] or to suppress tyrosinase by inhibiting p38 MAPK [73]. Additionally, MITF is suppressed through those signal pathways [29]. Besides the ERK or p38MAPK pathways, HSP70 is also reported to be involved in decreasing melanin [29]. Increased HSP70 expression leads to decreased UV-B induced skin pigmentation [29]. Transgenic mice expressing HSP70 exhibited decreased melanin deposition in the skin by UV radiation compared to wild-type animals [29]. HSP70 inhibits tyrosinase through MITF [29]. In the nucleus, HSP70 binds to MITF and inhibits the specific binding site of the tyrosinase gene promoter [29]. Ethanol extract of Arnica montana increases HSP70 activity and decreases tyrosinase activity and melanogenesis [74]. Although HSP70 directly decreased MITF through binding, it is also known that HSP70 modulates p53. HSP70 inhibits senescence by controlling p53 and cell cycle kinase Cdc2 [75]. Additionally, HSP70 deletion upregulates p53-dependent or ERK-dependent senescence pathways [76]. HSP70 suppresses JNK in cells stressed by heat [77,78]. JNK induces phosphorylation of p53, inhibiting p53 ubiquitination and degradation, and increasing p53 levels [79].

RF is electromagnetic energy that leads to molecular agitation in tissues and conducts electric current that is transformed into heat [43]. RF increases HSP47 and HSP70, which are involved in collagen synthesis [45,80]. HSP70 is increased by RF, and HSP70 modulates p53 and MITF. Thus, we hypothesized that RF could increase HSP70, which directly inhibits MITF. Furthermore, HSP70 could inhibit MITF by decreasing p53. Thus, decreased MITF and tyrosinase activity could decrease melanin synthesis.

We evaluated the effect of RF on decreasing melanin deposition with various combinations of RF settings (W and ms). Melanin content was not significantly decreased by RF doses of 50, 100, and 150 ms with 5 W. The melanin-decreasing effect was shown at doses of 100 ms/10 W, 150 ms/10 W, 50 ms/15 W, 100 ms/15 W, and 150 ms/15 W. However, the skin formed a crust at the RF doses of 150 ms/10 W, 50 ms/15 W, 100 ms/15 W, and 150 ms/15 W. Thus, an RF dose of 100 ms/10 W could decrease melanin deposition without skin irritation.

UV radiation induced the proliferation of keratinocytes by increasing the secretion of epidermal growth factor receptor, resulting in epidermal hyperplasia [81]. Moreover, chronic UV exposure to the skin increases melanocyte proliferation and density in the epidermis [82]. Our study also showed that cells positively stained by PCNA in the epidermis were increased by UV-B radiation. Those increased PCNA- positive cells were decreased by RF doses of 100 ms/10 W, 150 ms/10 W, 50 ms/15 W, 100 ms/15 W, and 150 ms/15 W. Additionally, we determined the effect of RF radiation on melanin synthesis with an in vitro model of α-MSH treated HEMn cells. RF decreased melanin content in the α-MSH treated HEMn cells 10 h after RF radiation. Melanosome maturation, which was evaluated by TEM, was also decreased by RF. After RF treatment, stage III or stage IV melanosome levels were decreased in the α-MSH treated HEMn cells. We evaluated whether RF changed HSP70 or p53 expressions in UV-B radiated skin. 

UV-B decreased the expression of HSP70, while it was increased by RF. UV-B increased the expression of p53, while it was decreased by RF. Moreover, UV-B increased MC1R and MITF, which are among the main signals of melanogenesis. However, RF also decreased those expressions in UV-B radiated skin. The expression of Rab32 and Rab27a, related to melanosome maturation [23], was also increased by UV-B radiation and decreased by RF. It seemed that RF could modulate HSP70 and p53, which decreased melanogenesis and melanosome maturation in our study. Next, we evaluated whether RF decreased expressions of various melanogenesis-induced signal pathways such as MC1R, CREB, and MITF through HSP70 or p53, using HSP70-overexpressed, HSP70-silenced, and p53-inhibited HEMn cells. Treatment with α-MSH decreased HSP70 expression and increased p53 expression. However, RF increased HSP70 expression and decreased p53 expression. Treatment with α-MSH increased expressions of MC1R, CREB MITF, RAB32 and RAB27a, but RF decreased those expressions in HEMn cells. The expressions of p53 also decreased under HSP70 overexpression. The expressions of MC1R, CREB, MITF, RAB32, and RAB27a were also decreased by HSP70 overexpression. The decreasing effects of RF on the expression of p53, MC1R, CREB, MITF, RAB32, and RAB27a were higher than those of HSP70 overexpression.

Next, we evaluated the effects of RF on the expression of p53, MC1R, CREB, and MITF in HSP70-silenced HEMn cells. When HSP70 was silenced, RF could not decrease the expressions of p53, MC1R, CREB, or MITF. Tyrosinase activity and melanin content were not decreased in the HSP70-silenced HEMn by RF.

When a p53 inhibitor was given to HEMn cells, the expressions of MC1R, CREB, and MITF were not decreased compared with α-MSH treated HEMn cells. However, RF led to decreased expression of MC1R, CREB, and MITF compared with α-MSH treated HEMn cells. The tyrosinase activity and melanin content were decreased by the p53 inhibitor. However, the decreasing effect was not higher than that of RF. It seemed that the inhibition of p53 could not decrease its down signaling of MC1R/CREB/MITF enough when α-MSH was given to HEMn cells, since the MC1R/CREB/MITF signaling pathway is directly upregulated by α-MSH. However, RF showed a more prominent decreasing effect of MC1R/CREB/MITF than p53 inhibition.

Skin pigmentation could increase by skin inflammation induced by various inflammatory cytokines, such as IL-1α, IL-6, COX-2, and TNF-α [83]. However, previous studies showed that RF radiation could decrease skin inflammation by decreasing inflammatory cytokines [40]. Thus, HSP70 or p53 modulation by RF is not the only mechanism for decreasing melanin synthesis by RF. However, our results indicate that RF could decrease melanin synthesis and melanosome maturation by modulating HSP70 and p53.

Since excessive skin pigmentation leads to cosmetic problems, there has been tremendous effort to develop skin pigmentation treatment with various drugs, cosmetics, chemical peeling, and laser treatments [84,85,86]. Even though hydroquinone has been used as a skin-whitening drug, it has various side effects, including burning, redness, and allergies [87]. Therefore, it has been of great interest for the cosmetic industry to find skin pigmentation treatments without complications. RF radiation has been used to increase collagen regeneration and decrease wrinkles without severe complications. Tissue overheating is rare with RF treatment, which causes skin irregularities in fewer than 0.08% of cases [43].

Furthermore, our study showed that RF could be used to attenuate skin pigmentation. In summary, RF decreased MC1R/MITF and tyrosinase activity by increasing HSP70 and decreasing p53, eventually decreasing melanin synthesis and melanosome maturation (Figure 5H).

## 4. Materials and Methods

### 4.1. Skin Pigmentation Model Induced UV-B Exposure and RF Application

HRM-2 mice (6 weeks old, male, 20–25 g), a hairless mouse capable of synthesizing melanin [88,89,90], were obtained from Japan SLC, Inc., (Shizuoka, Japan), and underwent acclimatization for 2 weeks. The mice were housed in cages under a 12 h light/dark cycle with a controlled temperature of about 23 °C, relative air humidity of about 50%, and ad libitum access to food and water.

After the adaptation period, the mice were randomly grouped into eleven groups as follows:

(1) Control (no exposure to UV-B with no irradiated RF).
(2) UV-B (exposure to UV-B at 200 mJ/cm^2^ with no irradiated RF).
(3) UV-B/RF 5 W/50 ms (exposure to UV-B/irradiated RF at 2 MHz, 5 Watt for 50 ms).
(4) UV-B/RF 5 W/100 ms (exposure to UV-B/irradiated RF at 2 MHz, 5 Watt for 100 ms).
(5) UV-B/RF 5 W/150 ms (exposure to UV-B/irradiated RF at 2 MHz, 5 Watt for 150 ms).
(6) UV-B/RF 10 W/50 ms (exposure to UV-B/irradiated RF at 2 MHz, 10 Watt for 50 ms).
(7) UV-B/RF 10 W/100 ms (exposure to UV-B/irradiated RF at 2 MHz, 10 Watt for 100 ms).
(8) UV-B/RF 10 W/150 ms (exposure to UV-B/irradiated RF at 2 MHz, 10 Watt for 150 ms).
(9) UV-B/RF 15 W/50 ms (exposure to UV-B/irradiated RF at 2 MHz, 15 Watt for 50 ms).
(10) UV-B/RF 15 W/100 ms (exposure to UV-B/irradiated RF at 2 MHz, 15 Watt for 100 ms).
(11) UV-B/RF 15 W/150 ms (exposure to UV-B/irradiated RF at 2 MHz, 15 att for 150 ms).

The mice were exposed to UV-B for 5 min once every 2 d for 10 d and then for 5 min every day for 3 d (total of 13 d) [91]. Subsequently, the mice were irradiated with RF and exposed to UV-B every 2 d for 28 d (Figure 1A).

This study was approved by the Center of Animal Care and Use ethical board of Gachon University (Approval Number LCDI-2018-0094) and executed in accordance with the Institutional Animal Care and Use Committee.

### 4.2. RF Irradiation System

The irradiation system (POTENZA, Jeisys Medical Inc., Seoul, Korea) used for this study was a bipolar pulse-type electrode array radiofrequency device. An impedance matching system was used to determine the compensation value by automatically measuring impedance, and RF was applied using a 16 ea (4 × 4) needle tip. RF was administered at 2 MHz using pulse-type, bipolar, alternating current oscillations in the animal experiment. Single pulse-type bipolar RF devices were used in the animal experiment and comprised an on-time pulse duration of 100 ms at a power density of 10 W/pulse. The invasive microneedle for RF application had a length of 13.6 mm, a diameter of 250 mm, and a needle-to-needle distance of 1.3 mm. Irradiation and treatment were performed with a disposable tip of 10 mm × 10 mm consisting of 16 electrodes. The tip was approved by NAMSA (Northwood, OH, USA) after biological compatibility testing.

### 4.3. In Vitro Model and RF Irradiation

Human primary epidermal melanocytes (HEMn; ATCC, Manassas, VA, USA) were grown in Dermal Cell Basal Medium (ATCC, Manassas, VA, USA) with a melanocyte growth kit (ATCC, Manassas, VA, USA). For establishing the in vitro model in HEMn, the cells were treated with 200 nM α-MSH (Sigma-Aldrich, St. Louis, MO, USA), and kept in an incubator at 37 °C in an atmosphere of 5% CO_2_ for 24 h. Then, the cells were irradiated with RF (2 MHz, 10 W for 100 ms), and incubated for 9, 10, 12, 24, and 48 h (Figure 2A).

### 4.4. Overexpression and Silencing of HSP70 and Treatment of p53 Inhibitor to the HEMn

Transfections with pcDNA3-HSP70-HA (Invitrogen, Carlsbad, CA, USA) and HSP70 shRNA (Santacruz biotechnology, Dallas, TX, USA) were performed using Lipofectamine 3000 reagent (Invitrogen, Carlsbad, CA, USA) according to the manufacturer’s protocol. After transfection, the cells were treated with 200 nM α-MSH) for 24 h. Then, the cells were irradiated with RF, and maintained for 48 h.

The p53 inhibitor (Nutlin-3; Cayman chemical, Ann arbor, MI, USA) of cultured cells was treated at 50 μM for 24 h. After 24 h, the cells were treated with 200 nM α-MSH for 24 h. Then, the HEMn cells were irradiated with RF and incubated for 48 h.

### 4.5. Measurement of Melanin Content in Cells

To assess melanin content in HEMn, the cells were seeded at 1 × 10^4^ cells/well in 96-well plates, and incubated for 24 h. After applying α-MSH and RF (after 9, 10, 12, 24 and 48 h), the cells were harvested by centrifugation at 12,000× *g* for 20 min and dissolved in 100 μL of 10% dimethyl sulfoxide (DMSO) and 1 N NaOH solution for 20 min at 95 °C. Absorbance at 490 nm was measured using a microplate reader (Molecular Devices, San Jose, CA, USA)).

### 4.6. Sample Preparation

#### 4.6.1. Extraction of RNA and cDNA Synthesis

The cells were homogenized by the RNAiso Plus reagent (Takara, Shiga, Japan) according to the manufacturer’s protocol.

The extracted RNA was quantified using the NanoDrop 2000 spectrophotometer (Thermo Fisher Scientific, Waltham, MA, USA), and converted to cDNA using a PrimeScript 1ST strand cDNA Synthesis Kit (Takara, Shiga, Japan) for quantitative real-time polymerase chain reaction (qRT-PCR).

#### 4.6.2. Paraffin-Embedded Tissue Sectioning

First, the skin tissues that were fixed by 4% paraformaldehyde (Sigma-Aldrich, St. Louis, MO, USA) were washed for 30 min for embedding. Next, skin paraffin blocks made using a tissue processor (Thermo Fisher Scientific, Waltham, MA, USA) were sectioned at 7 µm using a microtome (Leica, Wetzlar, Germany), and cooked at 37 °C overnight to keep them attached to the slides. The sectioned slides were passed through xylene and four concentrations of ethanol (100%, 95%, 80%, and 70%) to deparaffinate them for staining.

### 4.7. Quantitative Real-Time Polymerase Chain Reaction

For qRT-PCR, a reagent containing the SYBR Green reagent (Takawas mira) mixed with 1-µg of synthesized cDNA template, and a 10 pmol primer (Table S1), were dispensed into 384-well multi-plates. Then, the mixture was analyzed using the CFX386 Touch Real-Time PCR System (Bio-Rad, Hercules, CA, USA).

### 4.8. 3,3′-Diaminobenzidine Staining for Immunohistochemistry

The sectioned skin tissue slides were incubated in 3% hydrogen peroxide in methanol for 30 min at room temperature to block endogenous peroxidase. The tissue slides were washed using PBS and then incubated with primary antibodies (Table S2) in normal serum for 24 h at 4 °C. The slides were rinsed with PBS and incubated with a biotinylated secondary antibody using the ABC kit (Vector Laboratories Inc., Burlingame, CA, USA) for 2 h at room temperature. After washing with PBS, the tissue slides were developed using 3,3′-diaminobenzidine (Sigma-Aldrich) for 15 min to confirm the brown signal. To identify nuclei, tissue slides were stained in hematoxylin solution for 1 min, then mounted with a dibutylphthalate polystyrene xylene mounting solution (Sigma-Aldrich). Finally, images of the stained tissues were taken using an optical microscope (Olympus Optical Co., Tokyo, Japan), and analyzed using ImageJ software (NIH, Bethesda, MD, USA).

### 4.9. Transmission Electron Microscopy

Specimens were fixed for 12 h in 2% glutaraldehyde/2% paraformaldehyde in 0.1 M phosphate buffer (pH 7.4) and washed in 0.1 M phosphate buffer. Next, the specimens were post-fixed with 1% OsO_4_ in 0.1 M phosphate buffer for 2 h, dehydrated with an ascending ethanol series (50%, 60%, 70%, 80%, 90%, 95%, 100%, and 100%) for 10 min each, and infiltrated with propylene oxide for 10 min.

The fixed samples were embedded using a Poly/Bed 812 kit (Polysciences, Warrington, PA, USA), and polymerized in an electron microscope oven (DOSAKA, Katsumi, Japan) at 65 °C for 12 h. The block was equipped with a diamond knife in the ultramicrotome, cut into 200 nm sections, and stained with toluidine blue for optical microscopy.

The region of interest was then cut into 80 nm sections using the ultramicrotome, placed on copper grids, double stained with 3% uranyl acetate for 30 min and 3% lead citrate for 7 min, and observed under a TEM (JEOL, Tokyo, Japan) equipped with a Megaview III CCD camera (Soft Imaging System, Münster, Germany) at an acceleration voltage of 80 kV.

### 4.10. Fontana-Masson Staining

The skin tissues were incubated in Fontana ammoniacal silver solution (ScyTek, West Logan, UT, USA) overnight at room temperature, subsequently rinsed three times with distilled water, and then incubated in hypo solution for 3 min. Next, the tissues were washed in distilled water, counterstained with neutral red stain for 5 min, washed in distilled water, dehydrated in absolute alcohol, and mounted for observation.

### 4.11. Statistical Analysis

We performed Kruskal–Wallis tests to compare the three groups, followed by a Mann-Whitney *U* test as a post hoc test. This study was validated using an unpaired *t*-test. All results are presented as mean ± standard deviation, and the statistical analyses were performed using SPSS v.22 (IBM Corporation; Armonk, NY, USA).

## Data Availability

All data are contained within the article.

## Supplementary Materials

The following are available online, Figure S1. RF effect on decreasing melanogenesis in the p53-inhibited HEMn cells; Table S1: List of antibodies for immunohistochemistry; Table S2: List of primers for quantitative real time polymerase chain reaction.

## Sample Availability

All samples are available from the authors.
