# Supplementary material for: Attenuation Effect of Radiofrequency Irradiation on UV-B-Induced Skin Pigmentation by Decreasing Melanin Synthesis and through Upregulation of Heat Shock Protein 70
Source: Molecules. 2021 Dec 17;26(24):7648. doi: 10.3390/molecules26247648 (PMC8708156; doi:10.3390/molecules26247648)
Supplement: Supplementary file 1 [file molecules-26-07648-s001.zip › molecules-1496601-supplementary.pdf]

Supplementary figure

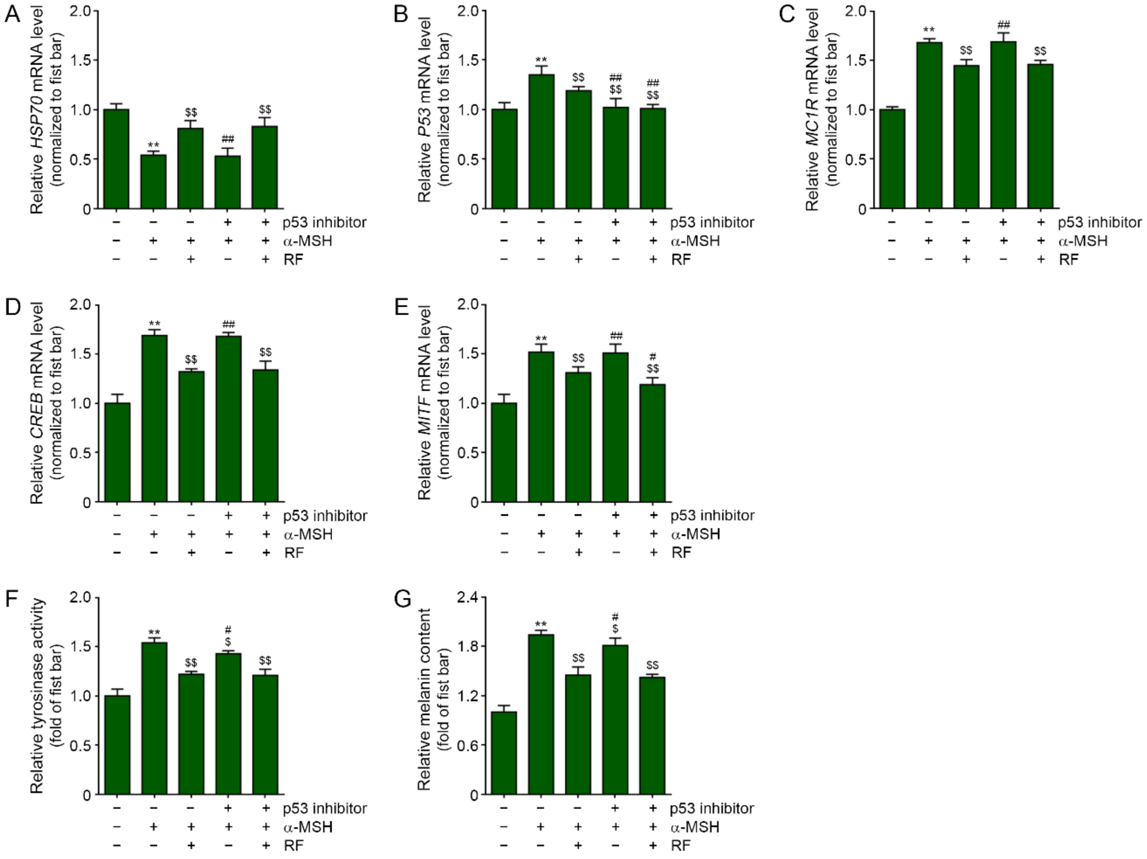

Figure S1. RF effect on decreasing melanogenesis in the p53-inhibited HEMn cells. (A–H) *HSP70* (A), *p53* (B), *MC1R* (C), *CREB* (D), and *MITF* (E) mRNA level of α-MSH and p53 inhibitor or untreated melanocytes were evaluated by qRT-PCR. (F) Relative tyrosinase activity of *α*-MSH and p53 inhibitor-treated melanocytes were evaluated by ELISA. (G) Relative melanin content of *α*-MSH and p53 inhibitor-treated melanocytes were evaluated by melanin content assay. HSP70, 70 kilodalton heat shock proteins; MC1R, melanocortin 1 receptor; CREB, cAMP-response element binding protein; MITF, microphthalmia-associated transcription factor; RF, radiofrequency; UV-B, ultraviolet-B; **, $p < 0.01$ vs. PBS; $, $p < 0.05$, $$, $p < 0.01$ vs. α-MSH/RF(-); #, $p < 0.05$, ##, $p < 0.01$ vs. α-MSH/RF(+).

Table S1: List of antibodies for immunohistochemistry

Antigen (host)	Company	Catalog no.	Dilution rate
HSP70 (Mouse)	Abcam	ab61097	1:500
p53 (Mouse)	Santa cruz biotechnology	sc-126	1:200
MC1R (Mouse)	LSBio	LS-C332324	1:500
MITF (Rabbit)	Cell signaling technology	LS-C117668	1:500
Rab32 (Mouse)	CSBio	LS-C204248	1:200
Rab27a (Rabbit)	Abcam	ab55667	1:200

Table S2: List of primer for quantitative real time polymerase chain reaction

Gene		Primers
ACTB	Forward	5'- GGGACCTGACTGACTACCTCAT -3′
	Reverse	5'- CCTTAATGTCACGCACGATTT -3′
HSP70	Forward	5'- TGAGGGTAAGATGATCATGCAG -3′
	Reverse	5'- ACATATTCCTCCACTGCGTTCT -3′
P53	Forward	5'- TGGATGACAGAAACACTTTTCG -3′
	Reverse	5'- GTGGATGGTGGTACAGTCAGAG -3′
MC1R	Forward	5'- CTTCCTGCATCTCACACTCATC -3′
	Reverse	5'- GGCGAGAAAGAGGTTGAAGTT -3′
CREB	Forward	5'- GCAGCTGTAACAGAAGCTGAAA -3′
	Reverse	5'- GGCATAGATACCTGGGCTAATG -3′
MITF	Forward	5'- AACCCCACCAAGTACCACATAC -3′
	Reverse	5'- GGACTTGGTTGGCATGTTTATT -3′
RAB32	Forward	5'- ACCACCAAAGCTTTCCTAATGA -3′
	Reverse	5'- ACACTGGGATTTGTTCTCTGCT -3′
RAB27a	Forward	5'- GACAAGTCCTGGATTCCTGAAG -3′
	Reverse	5'- CCCCTTTCTCCTTTTCTTCACT -3′
